# Development and initial validation of the determinants of physical activity questionnaire

**DOI:** 10.1186/1479-5868-10-74

**Published:** 2013-06-11

**Authors:** Natalie Taylor, Rebecca Lawton, Mark Conner

**Affiliations:** 1 Institute of Health Research, Bradford Royal Infirmary, Duckworth Lane, Bradford BD9 6RJ, UK; 2 Institute of Psychological Sciences, University of Leeds, Leeds LS2 9JT, UK

**Keywords:** Determinants, Barriers, Behaviour change, Theoretical domains framework, Intervention development

## Abstract

**Background:**

Physical activity interventions are more likely to be effective if they target causal determinants of behaviour change. Targeting requires accurate identification of specific theoretical determinants of physical activity. Two studies were undertaken to develop and validate the Determinants of Physical Activity Questionnaire.

**Methods:**

In Study 1, 832 male and female university staff and students were recruited from 49 universities across the UK and completed the 66-item measure, which is based on the Theoretical Domains Framework. Confirmatory factor analysis was undertaken on a calibration sample to generate the model, which resulted in a loss of 31 items. A validation sample was used to cross-validate the model. 20 new items were added and Study 2 tested the revised model in a sample of 466 male and female university students together with a physical activity measure.

**Results:**

The final model consisted of 11 factors and 34 items, and CFA produced a reasonable fit χ^2^ (472) = 852.3, p < .001, CFI = .933, SRMR = .105, RMSEA = .042 (CI = .037-.046), as well as generally acceptable levels of discriminant validity, internal consistency, and test-retest reliability. Eight subscales significantly differentiated between high and low exercisers, indicating that those who exercise less report more barriers for physical activity.

**Conclusions:**

A theoretically underpinned measure of determinants of physical activity has been developed with reasonable reliability and validity. Further work is required to test the measure amongst a more representative sample. This study provides an innovative approach to identifying potential barriers to physical activity. This approach illustrates a method for moving from diagnosing implementation difficulties to designing and evaluating interventions.

## Background

Physical activity interventions that target the general population may be useful [1,2]. However, there is evidence to support the success of tailored interventions to increase physical activity e.g., [3,4], especially when these interventions are tailored on the basis of theoretical constructs, such as attitudes, self-efficacy, or social support [5,6], rather than other factors such as age and sex. This suggests that tailored physical activity interventions that are theoretically informed may be particularly effective [7-10].

The application of theory within intervention studies lacks clarity, so that although physical activity interventions appear to have moderate sized effects [11], very little can be said about the role of theoretical components in producing these effects [12]. This is because too few interventions use a theoretical framework; less than half were found to do so in a study by Dombrowski, Sniehotta, Avenel, & Coyne [13], and there is often too little information about how a theory-based intervention has been developed [7]. Hence, links between interventions and theory-based determinants of behaviour change may be useful, as these can be examined in mediation analysis, thus helping to identify intervention effects [13].

Currently there are a number of theoretical models of behaviour that could be applied in this endeavour, such as the Health Belief Model HBM; [14], the Theory of Reasoned Action TRA; [15,16], and the Theory of Planned Behaviour TPB; [17,18], Social Cognitive Theory SCT; [19], many of which share common, or overlapping constructs, such as intention, social norms, beliefs, control/self-efficacy, etc. However, many of the theories used to inform behaviour change interventions are developed to understand (i.e. to explain or predict) behaviours [20], rather than to understand behaviour change, and the two are not entirely compatible [21]. This means that important constructs such as action planning e.g., [22], skills e.g., [23], or environment e.g., [24] may be overlooked by some theories.

A number of theoretical models and frameworks of behaviour change have been proposed for a range of behaviours, such as professional practice, addiction, and disease prevention e.g., [9,22,25-27], many of which include the assessment of barriers to change and subsequent tailoring of interventions [28]. Barriers hamper the implementation of behaviour change and have previously been classified as related to a number of factors including the individual (knowledge, skills, attitudes, habits), social context (influence of others), and environmental context (e.g., available resources, climate, etc.) [29].

The potential importance of barriers to change in the physical activity domain has been highlighted by several authors e.g., [30,31], and identification of such barriers can be seen to represent the common constructs from the aforementioned theories and frameworks of behaviour and behaviour change^a^. In recent work, attempts have been made to assimilate these common or overlapping constructs into a simple framework [9,23]. Michie et al. [7] proposed the theoretical domains framework (TDF) which contains 11 determinants of behaviour change (in addition to the nature of the behaviour), examples of which include ‘environment and resources’, ‘emotion’, ‘motivation and goals’, ‘beliefs about capabilities’, and ‘social influences’. Rather than a theoretical explanation of a set of behaviours identifying causal processes that link theoretical constructs, this pragmatic framework identifies key determinants and constructs, and provides a guide to relevant explanations of current behaviours which can be assessed and subsequently signal opportunities and methods for intervention [7]. Thus, accurate assessment of determinants of physical activity at the level of the individual has the potential to allow for tailored interventions that target those determinants representing a person’s barriers to physical activity. One way to achieve this level of assessment is via a questionnaire, but currently no such measure of behavioural determinants exists for physical activity.

Therefore the two studies reported here describe the development and initial validation of a Determinants of Physical Activity Questionnaire (DPAQ) and ask: 1) Does factor analysis of the measure support the specified model (convergent and discriminant validity)? 2) Does the measure demonstrate internal consistency and test-retest reliability? 3) Do scores on the different determinants differentiate between high and low exercisers (criterion validity)?

## Study 1

### Methods

#### Questionnaire development

Given that, a) frameworks of behaviour change are not stagnant and appear to be regularly updated [9,23,32], and b) the TDF does not claim to be a ‘theory’ containing causal processes that link constructs, it was deemed acceptable to modify the framework for the specific behaviour being studied. Therefore, determinant areas that did not fit with physical activity behaviour (i.e. social/professional role/identity, and memory, attention and decision processes) were omitted and additional determinant areas were considered. As there is recent evidence for the importance of ‘coping planning’ and ‘goal conflict’ for physical activity e.g., for coping planning: [33], e.g., for goal conflict: [34], it was decided that both would be considered as additional determinant areas. With nine determinants remaining from the TDF, the framework developed here for physical activity therefore consisted of 11 determinants in total.

The first phase of item development involved reviewing previous research on barriers to physical activity [35-46] so as to identify the key emotions, beliefs, control factors etc. These were then mapped onto the 11 theoretical determinants from the adapted TDF. Interview questions, previously formulated by Michie et al. were also used to inform item development as were the constructs underpinning each determinant area (e.g., for the determinant area ‘beliefs about consequences’, interview questions suggested included ‘what do they think will happen if they do X?’, and for the ‘emotion’ determinant, ‘does doing X evoke an emotional response?’). An example of this process is shown in Table 1 and the full mapping exercise is available from the first author.

**Table 1:** Beliefs about capabilities, constructs and barriers mapped onto this determinant

**Determinants**	**Constructs proposed by Michie et al. (2005)**	**Barriers**
Beliefs about capabilities	• Self-efficacy	• Not able to do PA
	• Control: behaviour/material/social environment.	• Cannot discipline myself to do PA
		• Face too many difficulties
	• Perceived competence	• Had problems in the past
	• Self-confidence	• Do not feel confident doing PA
	• PBC	• Lack persistence

Ultimately, six items were developed for each determinant area which reflected both the constructs making up each determinant and the specific barriers generated from the literature. Final amendments of item wording were made by the primary researcher (NT) based on feedback from the authoring team.

### Participants and procedure

Administrative employees from 49 universities across the UK distributed an email to staff and students, which contained information about the study and a link to the online version of the DPAQ. Ethical approval for the study was obtained from the Institute of Psychological Sciences Ethics Committee. As completing the questionnaire denoted consent (in accordance with the British Psychological Society ethical guidelines), no separate consent from participants was required. Participants were assured that their responses would be confidential and anonymous. Entry into a £100 prize draw was offered as an incentive for full questionnaire completion.

A total of 1463 staff and students visited the questionnaire website. Of these, 594 exited the site without providing any data. A further 37 completed less than half of the questionnaire and so were removed. Eight hundred and thirty two participants were included in the final dataset, of which 74.2% (*N* = 616) were female, 74.6% (*N =* 619) were White British, and for which the mean age was 33.6 (*SD* = 11.52; range = 18–70).

### Measures

An online version of the DPAQ was used to measure determinants of physical activity, for which each item was assessed using a 7-point scale ranging from 1 = *strongly disagree* to 7 = *strongly agree*. Example items include: ‘Nothing will get in the way of me doing physical activity’ to represent the ‘environmental context and resources’ determinant, and ‘I do not feel capable of doing physical activity’ to represent the ‘beliefs about capabilities’ determinant.

### Analysis

#### Confirmatory factor analysis

The aim here was to test the postulated 11 determinant structure, therefore confirmatory factor analysis (CFA) was used [47]. In the present study, a theoretical framework was being tested so it was deemed appropriate to use CFA as a model generating tool with the calibration sample and to subsequently use the validation sample in a strictly confirmatory sense to cross-validate the final model obtained [48,49].

### Discriminant validity and internal consistency reliability

A test of discriminant validity was undertaken to assess how distinct the constructs were from one another using formulae by Fornell & Larkner [50], and Anderson & Gerbing [51]. According to the Fornell & Larkner, two constructs display discriminant validity if the average of the estimate of variance extracted exceeds the square of the correlation between the two latent constructs. The second assessment was to determine whether the confidence interval around the correlation estimate between the two factors includes 1.0. The internal consistency of the scale was assessed using Cronbach’s alpha (α), with an alpha of ≥ .7 considered acceptable [52].

## Results

### Data screening

Missing values analysis (MVA) was undertaken on the final dataset (*N* = 832) and although it indicated that values were not missing completely at random (MAR) (*p* < .001), follow up tests revealed only .47% of the data was missing data and there were no variables whose pattern of missing values may have influenced the scale variables. As a result, expectation maximisation (EM) was used to impute missing values.

### Sample splitting

In order to cross-validate the proposed measure, the total sample was randomly split in half (with 416 cases in both the calibration and validation samples). The two samples did not significantly differ with regard to age, gender, ethnicity, and staff/student status (minimum *p* = .55).

### Descriptive statistics

The means, standard deviations, skewness and kurtosis values of the 11 subscales comprising the DPAQ for the calibration and validation samples indicated relatively high mean scores for six subscales (above 5 on a 7-point scale), but standard deviations indicated a sufficient amount of variance for each subscale^b^. Most items were negatively skewed but only one subscale (beliefs about consequences) was identified as extremely leptokurtic in the validation sample (2.2), indicating that scores were heavily concentrated around the mean. Mardia’s [53] coefficient of multivariate kurtosis (644.5, SE = 9.23) indicated multivariate normality was violated. Nevertheless, maximum likelihood estimation was used because it has previously resulted in accurate fit indices with ordered categorical data that were skewed and of varying degrees of kurtosis [54].

### Analysis

#### Confirmatory factor analysis

A full 11 factor model – including nine factors from the TDF and the two additional determinants ‘coping planning’ and ‘goal conflict’ - was specified and evaluated with the calibration sample using CFA, employing maximum likelihood estimation, in AMOS 19. There is widespread agreement that model fit should be examined using a range of acceptable fit indices [55]. The Comparative Fit Index (CFI) indicates how well the proposed model compares to the null model [56]. A cut-off point ≥ .95 for CFI indicates reasonable fit of the model. The Standardised Root Mean Square Residual SRMR; [57] is the square root of the average squared amount by which the sample variances and covariances differ from their estimates obtained under the assumption that the tested model is correct. The Root Mean Square Error of Approximation (RMSEA) measures the extent to which a model is supported per degree of freedom. Hu & Bentler [52] proposed that the SRMR and RMSEA should be below an upper boundary of .08 and .06, respectively, for adequate fit.

The data did not initially fit the model well, χ^2^ (2024) = 6158.18, *p* < .001, CFI = .655, SRMR = .225, RMSEA = .070 (CI = .068-.072). Upon inspection, modification indices (MIs)^c^, standardised residuals (SRs)^d^, and item content identified causes of model misspecification so it was decided to embark on post-hoc model fitting. For example, the largest MI was obtained for Sk4 (MI = 188.815), which also produced 5 standardised residuals below −2.58 and one above 2.58. Based on these results and after assessment of item content, Sk4 was removed. These changes subsequently improved the fit of the model χ^2^ (1960) = 5815.8, *p* < .001, CFI = .670, SRMR = .212, RMSEA = .069 (CI = .067-.071). Altogether, 38 amendments were made using these methods^e^. Therefore from this point forward, all specification and estimation with the calibration sample represent exploratory factor analysis (Byrne, 2001).

*The final model:* The original 11 factor model and remaining 31 items (Mardia coefficient = 214.0, SE = 4.44) fit the data to a satisfactory level, χ^2^ (379) = 757.2, *p* < .001, CFI = .921, SRMR = .121, RMSEA = .049 (CI = .044-.054). These results either fall within, or are approaching, acceptable CFA model fits.

### Discriminant validity and internal consistency reliability

After assessing all 55 pairs of factors using both methods, 52 achieved discriminant validity through either Fornell & Larkner [50] and/or Anderson & Gerbing [51] procedures ^f^. ‘Emotion’ – ‘motivation and goals’; ‘coping planning’ – ‘action planning’; and ‘coping planning’ – ‘goal conflict’ were the three pairs that did not reach the desired level. For each factor, internal consistency reliabilities were calculated using Cronbach’s alpha. Values ranged from α = .38 to .87; six of the subscales were approaching or exceeded .7, four were ≥ .6, and one subscale (social influences; .38) demonstrated unacceptable internal consistency^g^.

### Confirming the model using the validation sample

#### Confirmatory factor analysis

The 11-factor independent cluster model was tested using a strictly confirmatory approach with the validation sample (Mardia coefficient = 248.51, SE = 4.44). With the exception of the SRMR, the fit indices each fell within the acceptable levels, and therefore overall, the data supported the model, χ^2^ (379) = 793.3, *p* < .001, CFI = .909, SRMR = .122, RMSEA = .051 (CI = .046-.056).

### Discriminant validity and internal consistency reliability

Discriminant validity was achieved for all pairs of factors with the exception of the same three that did not reach the desired level in the calibration sample. Cronbach’s alphas ranged from α = .42 (social influences) to .85 (beliefs about capabilities). It is therefore apparent across both samples that ‘social influences’ consistently displayed weaknesses associated with an internal consistency measure of reliability.

### Discussion

The aim of this study was to develop and validate a self-report scale for determinants of physical activity based on the TDF, adapted for physical activity. A final 11 factor model containing 31 items resulted in an acceptable fit, which was confirmed using a validation sample. Five of the subscales achieved acceptable reliability and discriminant validity levels. However, it was clear from some fit statistics that the present model wasn’t wholly adequate. As such, further alterations were required to improve the measurement of some factors, and the overall fit of the model.

Therefore, Study 2 aimed to improve the convergent and discrminant validity, and internal consistency reliability, as well as to assess the criterion validity and test-retest reliability, of the DPAQ.

## Study 2

### Method

#### Participants and procedure

The data for this study were collected as part of a separate physical activity intervention study, whereby an administrative employee at a single large UK university distributed an email to approximately 8000 students living in university halls of residence. Information about the study and a link to the revised DPAQ was provided, for which entry into a cash prize draw was offered as an incentive for completion. Five hundred and forty three students were recruited and started the questionnaire (which equated to around a 6% response rate); sixty five did not go beyond demographic information and 13 did not provide enough information to be able to distribute the intervention so were excluded from the study, leaving N = 465 (86% of those who started the questionnaire). The sample population was predominantly female (f = 68.1%, m = 30%; five participants did not provide an answer) and white British (67.5%), with a mean age of 20.1 years (SD = 3.5).

### Materials

#### Determinants of physical activity

The revised version of the DPAQ was used to measure determinants of physical activity online. The questionnaire contained 31 of the original DPAQ items and 20 additional items, which were developed following assessment of additional literature e.g., [58-63] found for the revised six determinant areas. Table 2 presents a brief outline of the weaknesses identified for five subscales, alongside justifications for new item development and inclusion in the revised measure. Therefore a total of 51 items assessed the 11 subscales representing each determinant.

**Table 2:** Weaknesses and improvement plans associated with determinant areas

**Determinant area**	**Weaknesses**	**Improvement plans**
Environmental context and resources	Internal consistency reliability poor	Amended determinant label to represent ‘environmental resources’ only. Added 2 additional items to reflect accessibility of facilities, safety, and aesthetic factors
Skills	Internal consistency reliability poor	Added three items to encompass a broader range of skills associated with specific physical activities
Social influences	Internal consistency reliability poor	As descriptive norms, personal norms and perceived social support were represented in the final model under the same factor, four items representing these sub-areas of social influences were added to assess each type more accurately.
Beliefs about consequences	Internal consistency reliability poor	Included an item that assesses only ‘physical outcome expectations’ in order to avoid overlap with other determinants, such as ‘social influences’ and ‘beliefs about capabilities’. Included two items to assess perceptions of negative consequences of physical activity
Emotion	Discriminant validity with ‘motivation and goals’ poor	New items tap emotional antecedents of physical activity. Using ‘anxiety’, ‘stress’, ‘negative affect’, and ‘fear’ constructs outlined in the ‘emotion’ determinant from the Michie et al. framework, four items were developed to assess emotions that may influence a person’s decision to participate in physical activity.
Coping planning	Internal consistency reliability poor in sample 2, discriminant validity with ‘action planning’ and ‘goal conflict’ poor	The resulting three items represented proactive coping, reflective coping, and preventive coping. As these items were almost consistently reaching an acceptable level of reliability, and discriminant validity was questionable, three more items were developed to encompass proactive, reflective, and preventive coping, whilst attempting to distinguish them further from the action planning and goal conflict subscales.

### Physical activity

To examine the extent to which physical activity levels differed as a function of scores on the theoretical determinants, the Online Self-Reported Walking and Physical Activity Questionnaire (OSWEQ) was used, which has been validated against GTX3 accelerometers (for total energy expenditure: *r* = .611, *p* < .05) [64]. This measure was constructed online so that so that the type, frequency and time spent on each type of activity could be selected via drop-down boxes. This allowed for calculation of total energy expenditure (in METs) [65], representing walking and any other type of physical activity per day. Capturing this type of information enables types of activities performed to be distinguished from one another (e.g. walking, running, tennis, football, cleaning, etc.), and therefore should lead to more accurate calculations of energy expenditure, compared to other measures which do not specify types of activities e.g., [66,67].

### Data analysis

Analysis was undertaken in the same way as Study 1, with the addition of a 2 × 11 between subjects MANOVA to assess criterion validity (based on physical activity levels), and Pearson Product correlation, which was used to assess 14-day test-retest reliability on a subsample (*N* = 26). According to Cohen [68], *r* values of .1--3, .3--5, and .5--8 should be interpreted as small, medium, and large, respectively.

### Results

#### Descriptive statistics

MVA was undertaken on the dataset, for which Little’s MCAR test [69] was not significant (χ^2^ (854) = 860.80, *p* = .429), so EM was used to impute missing values.

The means, standard deviations, skewness and kurtosis values of the 11 subscales composing the revised DPAQ are displayed in Table 3. The mean scores (range = 3.32 – 6.06) for nine of the 11 determinant areas were around the mid-point, but ‘environmental resources’ and ‘beliefs about consequences’ were relatively high (above 5 on a 7 point scale – indicating that these determinants were least likely to represent barriers). Standard deviations ranged between .67 (beliefs about consequences) and 1.53 (beliefs about capabilities). Skewness and kurtosis values were low, with the exception of ‘beliefs about consequences’. Mardia’s [53] coefficient of multiavirate kurtosis (285.00, SE = 6.94) indicated multivariate normality was violated, so again, maximum likelihood estimation approach to CFA was used.

**Table 3:** Descriptive statistics of the 11 subscales composing the revised DPAQ (study 2)

**Subscale**	**Mean**	**SD**	**Skew**	**Kurtosis**
Knowledge	4.54	1.29	-.33	-.48
Environmental resources	5.37	1.14	−1.0	.84
Motivation and goals	4.80	1.17	-.25	-.45
Beliefs about capabilities	4.05	1.59	-.03	-.11
Emotion	4.94	1.20	-.55	-.31
Skills	4.65	1.26	-.34	-.60
Social influences	4.15	1.23	.00	-.64
Beliefs about consequences	6.06	.67	−1.0	1.44
Action planning	4.57	1.33	-.42	-.54
Coping planning	3.32	1.19	.41	-.18
Goal conflict	3.76	1.16	.09	-.40

### Analysis

#### Confirmatory factor analysis

Confirmatory factor analysis (CFA) was repeated to see if the data from the 51 items fit the 11-factor model. Although the data did not fit the model well, χ^2^ (1219) = 3258.97, *p* < .001, CFI = .782, SRMR = .140, RMSEA = .060 (CI = .058-.062), it was a better fit than the initial testing of the original model from Study 1. Inspection of MIs, SRs, and item content indicated causes of model misspecification so post-hoc model fitting was undertaken. These changes consisted of the removal of 17 items; Table 4 highlights the items that were retained as a result of this process.

**Table 4:** Revised DPAQ items retained following CFA (study 2)

**Determinant area**	**Items retained**
Knowledge	1. I know what the recommended levels of physical activity are (Kn1) *
	2. I DO NOT know the reasons why I should be meeting the nationally recommended PA guidelines (Kn2) *
	3. I have NOT previously read information about the current nationally recommended PA guidelines (Kn3) *
Environmental context and resources	1. Facilities are available to help me to do physical activity (En1) *
	2. There is NO WHERE to do physical activity near me (En2) *
	3. My local area is NOT very attractive and this puts me off doing physical activity (En4) **
Motivation and goals	1. I want to do physical activity (Mg1) *
	2. I CANNOT be bothered to do physical activity (Mg2) *
	3. I feel motivated to do physical activity (Mg3) *
Beliefs about capabilities	1. I DO NOT feel confident when doing physical activity (Bcap1) *
	2. Doing physical activity makes me feel embarrassed (Bcap2) *
	3. I FIND IT HARD to do physical activity when I see others doing well at physical activity (e.g. watching others run for a long time on the treadmill) (Bcap3) *
Skills	1. I can do physical activity to a good enough standard (Sk4) *
	2. I’ve NEVER really had sports skills so I DON’T do physical activity (Sk5) **
	3. I don’t seem to have the skills to keep going in physical activity sessions (Sk6) **
Emotion	1. Daily life is too stressful for physical activity (Em4) **
	2. I have too many negative emotions which prevent me from doing physical activity (Em5) **
	3. When I think about doing physical activity, I start to worry (Em6) **
Social influences	1. My friends DON’T support or encourage my physical activity (Si3)**
	2. The people I spend my free time with don’t do physical activity (Si6) **
	3. I DON’T have anyone to do physical activity with (Si7)**
Beliefs about consequences	1. If I do PA, it will benefit me in the short term (e.g. burn calories, sleep better etc.) (Bco1)*
	2. If I do PA it will benefit me in the long term (e.g. live longer, lose weight etc.) (Bco2)*
	3. I think physical activity will change my life for the better (Bcon4) **
Action planning	1. I tend to plan where my PA will happen (e.g. at the park, leisure centre etc.) (Ap2)*
	2. I do not tend to plan when my PA will happen (e.g. Monday at 6pm etc.) (Ap3)*
	3. I tend to plan how my PA will happen (e.g. how to get there, kit needed etc.) (Ap4)*
	4. I do not tend to plan what type of PA I will do (e.g. aerobics class, walking to work, session at the gym etc.) (Ap5)*
Coping planning	1. I know what to do in difficult situations in order to make sure I do the physical activity I have planned (Cp2) **
	2. I get easily distracted from the physical activity I have planned (Cp5) **
	3. I always work around obstacles to physical activity; nothing really stops me (Cp6) **
Goal conflict	1. I WOULD NOT be prepared to give up work ambitions to do physical activity (Gc1) *
	2. I would be prepared to give up things I usually do in my leisure time for physical activity (Gc2) *
	3. I WOULD NOT be prepared to give up spending time with my friends for physical activity (Gc3) *

* Items from the original DPAQ version.

** Items added after CFA undertaken in Study 1 and tested and Study 2.

The final model. The final model (Mardia coefficient = 142.54, SE = 4.6) consisted of 11 factors and 34 items and fit the data to a reasonable level, χ^2^ (472) = 852.3, p < .001, CFI = .933, SRMR = .105, RMSEA = .042 (.037-.046), with all fit indices falling within, or approaching, acceptable CFA model fits, and comparing well to other validated psychometric questionnaires e.g., [70-72]. Item loadings ranged from .43 to .90 (Table 5), and factor correlations ranged from .10 to .82. All pairs of factors achieved discriminant validity, and nine factors were approaching or exceeded an internal consistency of α = .7, with a noticeable improvement in ‘social influences’; ‘beliefs about consequences’ (α = .57) and ‘knowledge’ demonstrated less internal consistency reliability (α = .64)^h^. Positive and significant correlations for all 11 determinants, ranging between *r* = .45 (p < .05) and *r* = .91 (p < .01), indicated acceptable to strong levels of test-retest reliability over 14 days (Table 6).

**Table 5:** Item loadings and error terms for the 11 determinants from the revised DPAQ (study 2)

**Subscale**	**Item**	**Item loading**	**Error term**
Knowledge	Kn1	.579	.34
	Kn2	.591	.35
	Kn3	.676	.46
Environment	En1	.760	.58
	En2	.768	.59
	En4	.426	.18
Motivation and goals	Mg1	.465	.22
	Mg2	.742	.55
	Mg3	.756	.57
Beliefs about capabilities	Bcap1	.903	.82
	Bcap2	.869	.76
	Bcap3	.673	.45
Skills	Sk4	.672	.45
	Sk5	.725	.53
	Sk6	.787	.62
Emotion	Em4	.439	.62
	Em5	.789	.59
	Em6	.765	.19
Social influences	Si3	.599	.43
	Si6	.639	.36
	Si7	.654	.41
Beliefs about consequences	Bcon1	.437	.19
	Bcon2	.611	.37
	Bcon4	.661	.44
Action planning	Ap1	.784	.61
	Ap2	.718	.52
	Ap3	.836	.70
	Ap4	.773	.60
Coping planning	Cp2	.613	.38
	Cp5	.730	.53
	Cp6	.750	.56
Goal conflict	Gc1	.480	.23
	Gc2	.707	.50
	Gc3	.687	.47

**Table 6:** Cronbach’s alpha, test-retest reliabilities, and differences in subscale scores between low and high exercisers for the revised DPAQ (study 2)

**Determinant area**			**<900 mets**		**>900 mets**	
	**α**	***r***	**M**	**SD**	**M**	**SD**
Knowledge	.64	.45*	4.4_a_	1.3	4.6_a_	1.3
Environmental context	.67	.71**	4.9_a_	1.0	5.4_b_	1.1
Motivation and goals	.69	.79**	4.4_a_	1.2	4.9_b_	1.1
Beliefs about capabilities	.85	.86**	3.5_a_	1.6	4.1_b_	1.6
Skills	.77	.87**	4.6_a_	1.1	5.0_b_	1.2
Emotion	.70	.91**	4.0_a_	1.2	4.7_b_	1.2
Social influences	.66	.82**	3.7_a_	1.2	4.2_b_	1.2
Beliefs about consequences	.57	.63**	6.1_a_	.6	6.1_a_	.7
Action planning	.86	88**	4.0_a_	1.2	4.7_b_	1.3
Coping planning	.73	.78**	2.9_a_	1.0	3.4_b_	1.2
Goal conflict	.65	82**	3.6_a_	1.2	3.8_a_	1.2

Note. For the univariate ANOVA analysis comparing determinant scores for high and low exercisers, means in the same row that do not share the same subscript differ at the *p* < .05 level.

*p < .05; **p < .01.

### Criterion validity

The difference in DPAQ subscale scores between individuals performing over the recommended PA levels (> 900 METs; high exercisers), and those either just meeting or failing to meet the guidelines (< 900 METs; low exercisers) was assessed using an 11 × 2 between subjects MANOVA (Table 6). Across determinants, there was a significant difference in scores on the DPAQ between low (*N =* 66) and high exercisers (*N* = 400), *F*(11, 466) = 2.90, *p* < .01, although this difference was modest, partial η^2^ = .07, which may be partly explained by the low *N* in the ‘low exerciser’ group.

The univariate ANOVAs showed a significant difference between high and low exercisers for all determinants, except ‘knowledge’, ‘beliefs about consequences’, and ‘goal conflict’, but means for all eleven determinants were lower for the low exercisers, indicating low exercisers reported more barriers as they were further away from the optimal score on each subscale.

### Discussion

The aim of Study 2 was to test a revised version of the DPAQ using CFA. The final 11 factor model contained 34 items and resulted in a reasonable fit, demonstrating improvement in the overall fit statistics, discriminant validity, and internal consistency reliability compared to the earlier version of the DPAQ. Test-retest reliability was additionally assessed and the measure presented a desirable level of consistency over a 14-day period. In total, 17 items were discarded; of the 31 items which were retained during the initial modelling process, 21 remained, and of the 20 new items added in the previous remodelling phase, 13 were retained. All determinant areas consisted of three items, with the exception of ‘action planning’, which contained four. When tested for criterion validity, eight of the subscales significantly differentiated between high and low exercisers, with ‘emotion’ and ‘action planning’ showing the greatest differentiation, indicating that it might be appropriate to target low exercisers with interventions to address these areas.

Limitations of study 2 include the inability of the DPAQ to differentiate between high and low exercisers for some subscales. For ‘goal conflict’, it may be that this is a perceived barrier for most individuals as people regularly pursue multiple goals simultaneously [34], and this may also be a reason for why this subscale achieved the lowest scores (therefore representing a high barrier) out of all the determinants for both subgroups. These results suggest that an intervention which aims to address this particular determinant area may help to increase physical activity levels of university students who are exercising both above and below the recommended guidelines.

For the other two subscales that did not distinguish between high and low exercisers (‘knowledge’ and ‘beliefs about consequences’), the scores for both groups were relatively high, which is unsurprising given the capability of mass media campaigns to reach out to undifferentiated national audiences regarding information and outcomes associated with physical activity e.g., [73,74]. The high scores on these subscales may also suggest that possessing such information may not be enough to induce activity in low exercisers, especially if they perceive other predominant barriers (e.g. low beliefs about capabilities, or low motivation). This supports the idea that assessing determinants on an individual basis to identify which barriers are prominent for different people may be beneficial before providing an intervention that aims to help increase physical activity. Furthermore, these results may also imply that providing interventions to tackle these two areas might not be as effective as those aimed at other determinant areas with a university student population, but this is something that should be tested.

## General discussion

Based on a theoretical framework of behaviour change, 11 scales measuring the psycho-social determinants of physical activity in university staff and students were developed, tested, validated, revised and re-tested. This resulted in good discriminant validity and test-retest reliability, and reasonable to good internal consistency for the majority of determinant areas. Eight of the subscales in the final model also demonstrated criterion validity. Each significantly differentiated between high and low exercisers, indicating that individuals undertaking no, or minimal physical activity report stronger barriers in these areas than those who exercise over the recommended levels.

The DPAQ identifies nine of the 11 theoretical determinants from the TDF as relevant to physical activity behaviour. However, attempting to substitute established theories of behaviour with a theoretical determinants approach in order to facilitate behaviour change does not come without problems. First, unlike the operation of theory, the framework does not specify relationships between each of the determinant areas. For example, the Theory of Planned Behaviour [17,74] specifies that intention is directly related to behaviour, but that the relationships between behaviour and the other constructs in the model are mediated partly or fully through intention. By contrast, such patterns are not identified by the theoretical determinants approach, and thus it is not an attempt to replace such theories [75].

Despite demonstrating some promising reliable and valid properties for the DPAQ, this study has four key limitations. First, in study 1, administrative employees at 49 universities were asked to distribute the recruitment email; although it was possible confirm the number of people who visited the site versus completed the questionnaire, we were unable to state how many students received this email and therefore cannot provide an approximate response rate. However, for study 2, we improved our recruitment method to ensure we could provide an approximate response rate, which was poor (6%). Second, both studies relied on voluntary samples of university staff and students, who were predominantly female (71% across both studies), and the Study 1 sample was split for model building and validation purposes, thus the results obtained here may not be generalisable to the wider population. Future work should incorporate a recruitment strategy that enhances the likelihood of recruiting a sample more evenly balanced with respect to gender, increases the response rate, and uses independent samples to confirm current findings. Third, in study two, 17 items were removed from the original questionnaire, which could have resulted in a sample specific model fit. As such, a separate sample should be used to validate the final model. Fourth, this research could be improved by using an objective measure of physical activity to assess criterion validity, given this mode of assessment is accepted as more valid and reliable than that of self-report [76,77].

In this paper we report the development of the DPAQ – a novel measure of determinants of physical activity. The next stage, which will allow us to make claims about the power of the determinant framework as a basis for behaviour change, is to target specific determinants to assess whether this a) changes the reports of the determinant area [78,79], and b) changes behaviour [13]. To design physical activity interventions to discover which determinants can serve as vehicles of physical activity behaviour change, as well as specifying which determinant-specific strategies are effective in helping to produce the desired change [7,80], may well be the next logical step in taking this work forward. Work to test this idea is currently underway.

Whilst this research has demonstrated that the DPAQ is able to identify that lower exercisers report stronger barriers to physical activity, information about whether targeting determinants that represent predominant barriers for individuals through a tailored intervention can improve physical activity is also required. The DPAQ could therefore be the tool used to allocate matched interventions to tackle determinant areas representing high barriers to physical activity, which could be tested against the effectiveness of miss-matched interventions tackling determinants representing low barriers.

Finally, although the DPAQ has been developed for physical activity behaviour, this questionnaire also has the potential to be adapted for use in other health domains to allow for the tailoring of interventions for behaviours such as healthy eating, screening, smoking, etc. In support of this suggestion, the need for such a measure has been highlighted [29], so as to strengthen approaches to improve the performance of health professionals by tailoring interventions to identified barriers. However, depending on the behaviour targeted, it may be necessary to include specific behaviour-relevant constructs, for example habit/addiction for measures developed to address smoking/drugs, or to return to the original determinant areas from the TDF for improving performance in the health practitioner domain. The development process of the DPAQ has highlighted that it is possible to adapt and use the TDF with a degree of flexibility to identify determinants of behaviour change through a questionnaire.

## Conclusions

Clearly, more research should be undertaken to fully understand the uses and limitations of the DPAQ, but after two validation studies, it is believed that a measure of the determinants of physical activity has been developed, which demonstrates promising reliability and validity. Providing the 34 item questionnaire is validated on a separate sample, these findings should provide sufficient support to justify the use of this measure to assess determinants of physical activity in university staff and students, and to allocate interventions to individuals based on these subscale scores. Consequently, it is recommended that the DPAQ be tested in this manner to provide information about how changeable these determinants are, if changes in determinant areas directly influence physical activity behaviour, and finally whether targeting determinants that represent barriers for individuals through a tailored intervention can improve physical activity.

## Availability of supporting data

The data set(s) supporting the results of this article are available in the Essex ZendTo repository, [unique identifier: 7136; hyperlink: http://www.esds.ac.uk/Lucene/Search.aspx].

## Endnotes

^a^For example, not feeling confident about physical activity may be represented by the self-efficacy construct, or not feeling motivated to do physical activity may be represented by the intention construct, both of which can be found in a number of social-cognitive theories of behaviour e.g., [22,25,38].

^b^Descriptive statistics are available on request from NT.

^c^MIs were provided by AMOS for all parameters constrained to zero and indicate when an item may cross load or load onto a different factor [67].

^d^The standardised residual matrix identifies pairs of items that are either under or over-predicted by the model [68], for which values > +/−2.58 are considered to be large [70].

^e^Items subsequently retained and discarded are available on request from NT.

^f^According to the Fornell & Larkner (1981) test, two constructs display discriminate validity if the average of the estimate of variance extracted exceeds the square of the correlation between the two latent constructs. The second assessment was to determine whether the confidence interval around the correlation estimate between the two factors includes 1.0 [59].

^g^Values for all subscales from both samples are available on request from NT.

^h^Factor correlations, and discriminant validity values for all subscales are available on request from NT.

## Abbreviations

DPAQ: Determinants of physical activity questionnaire; OSWEQ: Online Self-reported walking and exercise questionnaire; DoH: Department of health; CFA: Confirmatory factor analysis; SRMR: Standardised root mean square residual; CFI: Comparative fit index; RMSEA: Root mean square error of approximation; GFI: Goodness of fit index; TDF: Theoretical domains framework; EM: Estimation maximisation; MVA: Missing values analysis.

## Competing interests

The authors declare that they have no competing interests.

## Authors’ contributions

NT led the design and coordination of the study, performed the statistical analysis, and led the writing process. RL participated in the design of the study, advised on questionnaire item content and analysis, and helped to draft the manuscript. MC participated in the design of the study, advised on questionnaire item content and analysis, and helped to draft the manuscript. All authors read and approved the final manuscript.

## Authors’ information

NT, RL, and MC have previously (and currently) worked on projects that involve using the TDF to identify barriers and design interventions using theoretically underpinned behaviour change techniques to design tailored interventions to address key barriers for a range of health behaviours. The paper that follows this demonstrates 1) the use of the questionnaire as a tool to identify key barriers, and 2) the effect of interventions tailored to key barriers to undertaking physical activity using a matched-mismatched randomised controlled trial design.
